# Characterization of brain mGluR5 binding in a pilot study of late-life major depressive disorder using positron emission tomography and [^11^C]ABP688

**DOI:** 10.1038/tp.2015.189

**Published:** 2015-12-08

**Authors:** C DeLorenzo, J Sovago, J Gardus, J Xu, J Yang, R Behrje, J S D Kumar, D P Devanand, G H Pelton, C A Mathis, N S Mason, B Gomez-Mancilla, H Aizenstein, J J Mann, R V Parsey

**Affiliations:** 1 Department of Psychiatry, Columbia University, New York, NY, USA; 2 Department of Psychiatry, Stony Brook University, Stony Brook, NY, USA; 3 Novartis Institute for BioMedical Research, Novartis Pharma AG, Basel, Switzerland; 4 Department of Applied Mathematics and Statistics, Stony Brook University, Stony Brook, NY, USA; 5 Department of Preventive Medicine, Stony Brook University, Stony Brook, NY, USA; 6 Novartis Pharmaceuticals Corporations, East Hanover, NJ, USA; 7 Department of Radiology, University of Pittsburgh, Pittsburgh, PA, USA

## Abstract

The metabotropic glutamate receptor subtype 5 (mGluR5) has been implicated in the pathophysiology of mood and anxiety disorders and is a potential treatment target in major depressive disorder (MDD). This study compared brain mGluR5 binding in elderly patients suffering from MDD with that in elderly healthy volunteers using positron emission tomography (PET) and [^11^C]ABP688. Twenty elderly (mean age: 63.0±6.3) subjects with MDD and twenty-two healthy volunteers in the same age range (mean age: 66.4±7.3) were examined with PET after a single bolus injection of [^11^C]ABP688, with many receiving arterial sampling. PET images were analyzed on a region of interest and a voxel level to compare mGluR5 binding in the brain between the two groups. Differences in [^11^C]ABP688 binding between patients with early- and late-onset depression were also assessed. In contrast to a previously published report in a younger cohort, no significant difference in [^11^C]ABP688 binding was observed between elderly subjects with MDD and healthy volunteers. [^11^C]ABP688 binding was also similar between subgroups with early- or late-onset depression. We believe this is the first study to examine mGluR5 expression in depression in the elderly. Although future work is required, results suggest potential differences in the pathophysiology of elderly depression versus depression earlier in life.

## Introduction

As the population ages, focus is increasing on management of late-life depression.^1^ Major depressive disorder (MDD) is common in older populations worldwide and can decrease the quality of life, as well as worsen medical outcomes, decrease physical, cognitive and social functioning, and increase risk of suicide.^1, 2, 3^ Although estimates of prevalence vary based on diagnostic criteria used and characteristics of the population under study, MDD rates as high as 42% have been reported in older adults.^2, 3^

Multiple studies have explored differences in MDD symptoms between adult and elderly populations, as well as the variable behavioral characteristics associated with early- and late-onset depression in the elderly.^4, 5^ A recent study has also evaluated structural brain changes associated with depression and Alzheimer's disease in the elderly.^6^ Yet, little is known about neurochemical differences that may exist in elderly depression. Therefore, to aid in treatment and potentially prevention, it is necessary to explore depression pathophysiology in elderly individuals.

Metabotropic glutamate receptors (mGluRs) have been implicated in the underlying pathology of a number of neurological and psychiatric disorders, including MDD.^7^ In particular, animal data suggest that one such receptor, the mGluR subtype 5 (mGluR5), may be a potential target for the treatment of anxiety-related and affective disorders.^7^ Preclinical studies showed that mGluR5 antagonists can promote an antidepressant response in rodents^8, 9, 10, 11, 12^ and that mGluR5 knockout mice exhibit antidepressant-like behavior.^12^ Lower mGluR5 density has also recently been reported in the brain of individuals with MDD (average age: 40.8 years) compared with healthy volunteers.^13^ Further, using positron emission tomography (PET), two rapid antidepressant strategies, sleep deprivation (wake therapy) and infusion of ketamine, have been shown to induce changes in binding at mGluR5, implicating this receptor in their mechanism of action.^14, 15^

Despite diverse evidence linking mGluR5 to MDD and antidepressant action, only a handful of studies have evaluated this receptor in aging/the elderly. A cognitive aging study in rats assessed spatial-memory faculties. Using quantitative immunofluorescence imaging of excised hippocampal sections from aged and young control rats, the study found comparable hippocampal mGluR5 levels in unimpaired aged and young rats, whereas impaired aged rats had only 56% of the level of young rats.^16^ Another study probed striatal mGluR5 densities in 3- and 24-month-old rats. Using western blotting and chemiluminescence, a 28.8% lower striatal mGluR5 was found in the elder rodents.^17^ In humans, a post-mortem study evaluated mGluR5 in elderly participants without disease by quantifying the percentage of mGluR5-expressing neurons in the caudate nucleus. In young subjects (*n*=3, average age=30.3), 40% of caudate nucleus neurons expressed mGluR5 receptors, compared with 80% in elderly subjects (*n*=6, average age=78.2).^18^ However, a recent *in vivo* PET study reported no statistically significant association between age and [^11^C]ABP688 binding.^19^ To our knowledge, no study has examined mGluR5 in elderly depression.

In this pilot study, [^11^C]ABP688 and PET were used to assess regional differences in mGluR5 binding in the brain of elderly patients with MDD compared with elderly healthy volunteers. ABP688 is an mGluR5 antagonist that binds to an allosteric site of the receptor with high affinity and high specificity.^20^ Preclinical studies in rats,^20, 21^ baboons^22^ and results from the recent PET studies in humans^13^ demonstrate that [^11^C]ABP688 is suitable to measure mGluR5 availability in the brain. Differences in [^11^C]ABP688 binding between patients with early- and late-onset depression were also assessed.

## Materials and methods

### Study design

This was a pilot study designed to estimate regional distributions of mGluR5 in the brain (CABP688A2102; ClinicalTrials.gov identifier: NCT01528241). The study was conducted at the following two centers: Columbia University, New York, NY, USA and the University of Pittsburgh, Pittsburgh, PA, USA.

### Participants

Male and female subjects aged 55–80 years with and without MDD were eligible to participate in this study. Twenty elderly (mean age: 63.0±6.3; 11 recruited at the Columbia University) subjects with MDD and 22 elderly (mean age: 66.4±7.3; 12 recruited at the Columbia University) healthy volunteers completed the study. All subjects were required to be non- or light-smokers, consuming six or fewer cups of coffee a day, and in good general health as determined by past medical history and physical examination, vital signs, electrocardiogram and laboratory tests at screening. (If the lab values were within 10% of normal range and are not deemed clinically significant by senior clinical staff and the subject was otherwise in healthy condition, the subject was included in the study.) Subjects could not have any surgical or medical condition, which might significantly alter the distribution, metabolism or excretion of [^11^C]ABP688 or which may jeopardize the subject in case of participation in the study. Other general exclusion criteria consisted of the following: use of psychotropic prescription drugs within 2 weeks before the PET scan or use of other prescribed drugs or over-the-counter medication if deemed to affect study results; presence and/or history of a clinically significant major neurological or psychiatric (for controls) disorder; presence and/or history of symptoms consistent with mild cognitive impairment or dementia as evidenced by either a Montreal Cognitive Assessment score of 25 or less or a Mini-Mental State Examination (MMSE) score of 26 or less; or any significant illness within 2 weeks before imaging.

Diagnosis of MDD, either a single episode or recurrent, was based on Diagnostic and Statistical Manual of Mental Disorders, Fourth Edition (DSM-IV) criteria and was established by the Structured Clinical Interview for DSM-IV (SCID). Patients were required to score at least 16 on the 17-item Hamilton Depression Rating Scale (HAM-D) and at least 4 (moderately ill) on the Clinical Global Impression Scale (CGI-S).

Exclusion criteria specific to MDD subjects included the following: presence and/or history of clinically significant major psychiatric disorder, other than MDD or generalized anxiety disorder; active suicidal ideation and plan; responding satisfactorily to antidepressant or antianxiety treatment or inability to tolerate medication washout. Subjects receiving benefit from antidepressant medications were not enrolled. If the current medication regiment failed, subjects were washed off of these medications, under the supervision of a clinician.

All participants provided written informed consent before study participation. The study was conducted in compliance with the Good Clinical Practice guidelines and was approved by the Ethics Committee/Institutional Review Board of both participating centers.

### PET and MRI examinations

[^11^C]ABP688 was prepared as previously described.^23^ A single bolus injection was administered to each subject, with administered radioactivity between 173 and 630 MBq. The mean (s.d.) administered activity of [^11^C]ABP688 was 368.81 (84.51) MBq and 387.54 (112.92) MBq for healthy volunteers and patients with MDD (*P=*0.54), respectively. The specific radioactivity of [^11^C]ABP688 was 16–175 GBq μmol^−1^. The mean (s.d.) specific activity was 53.27 (38.53) GBq μmol^−1^ and 60.63 (38.93) GBq μmol^−1^ for healthy volunteers and patients with MDD (*P=*0.54), respectively. Each injection was ~10 ml containing between 0.34 and 5.45 μg of ABP688. The range of injected doses and/or specific activities should not affect binding, as there was no observed relationship between average activity and either of these quantities. The mean (s.d.) injected mass was 2.31 (1.20) μg and 2.21 (1.41) μg for healthy volunteers and patients with MDD (*P*=0.80), respectively.

PET imaging was performed using an ECAT EXACT HR+ (Siemens/CTI, Knoxville, TN, USA) system at both sites. Following a 10-min transmission scan for attenuation correction, [^11^C]ABP688 was administered and emission data were collected for 60 min (10 × 1 min and 10 × 5 min frames). PET images were reconstructed using filtered back-projection, as previously described.^23^

All subjects received a magnetic resonance imaging (MRI) scan for anatomical reference and to exclude any structural brain abnormalities. MRI images were acquired on a Siemens 3T scanner (University of Pittsburgh, repetition time (TR), 2200 ms; echo time (TE), 3.43 ms; flip angle (FA), 9° matrix, 256 × 192; voxel size, 1 mm isotropic) and a GE 3T Signa HDx system (Columbia University, TR, 7.344 ms; TE, 2.988 ms; FA, 9° matrix, 256 × 256; voxel size, 1 mm isotropic).

Use of the same PET scanner at each site allowed comparable PET-based measurements. Although the MRI scanner was different, MRIs were solely used for anatomical delineation, and with the same resolution, scanner differences were unlikely to affect outcome.

### Blood sampling and metabolite measurements

Before study initiation, both sites standardized methods to ensure that blood acquisition and analysis occurred identically across sites. If possible, a catheter was inserted in the radial artery for blood sampling. Arterial samples were collected automatically within the first 4 min and manually thereafter (Columbia University) or entirely manually (University of Pittsburgh).

Authentic tracer and labeled metabolites in the plasma were separated using Waters Sep-Pak tC_18_ cartridges as described.^24^ Unmetabolized parent fraction levels were fit with a Hills function.^25^ The input function was calculated as the product of the interpolated parent fraction and the total plasma counts. These combined data were then fit as the combination of a straight line and the sum of three exponentials, describing the function before and after the peak, respectively.

For five subjects with plasma data, metabolite data were not available; therefore, an analytical method was implemented to substitute for the missing data, similar to previous studies.^26, 27, 28, 29^ Average arterial metabolite (population) curves were used to fit the standard two-tissue compartment model (2TCM).^30^ For comparison purposes, the population curve was also applied to subjects with complete metabolite data, and percentage difference between results obtained with individual and population curves calculated. Comparison of imaging results (binding potential relative to the non-displaceable compartment) with population and individual metabolite curves showed an average absolute percent difference ranging between 3.68±6.04% (dorsal putamen) and 8.76±25.04% (medial prefrontal cortex).

### Image analysis

To standardize study results, all images were analyzed by a single center (Columbia University) regardless of location of acquisition by image analysts who were blinded to diagnosis, using MATLAB (The MathWorks, Natick, MA, USA). Motion correction, co-registration to the MRI and automatic, atlas-based regional delineation of the MRI were performed as previously described.^23^ All processing steps were visually inspected for accuracy, or manually corrected as needed, before PET-binding analysis.

For subjects with arterial blood sampling (and either individual or population-based metabolite values), the standard 2TCM^30^ was used to calculate the total volume of distribution (*V*_T_: ratio of the concentration of the ligand in the region of interest to that in the plasma at equilibrium^30^) in each region of interest. S.e. values of the *V*_T_ measurement were computed using a bootstrap algorithm that takes into account errors in metabolite, plasma and brain data.^31^ To compare *V*_T_ to binding estimated in the absence of blood sampling, distribution volume ratio (DVR) was calculated by dividing regional *V*_T_ with *V*_T_ in the gray matter of the cerebellum.

An *in vitro* receptor-binding study showed that mGluR5s were present in the cerebellum in rat, rhesus monkey and human brain.^32^ Further examination using blocking experiments in baboons suggests that ~30% of cerebellar binding is specific binding, and that binding in the cerebellar gray matter may be lower than that of the full cerebellum.^14^ Therefore, the gray matter of the cerebellum was chosen as the most suitable reference region in humans. A brain region devoid of specific binding was not identified, and hence an ideal reference region for [^11^C]ABP688 has not been identified. Despite this, DVR was successfully used in previous clinical PET studies to quantify mGluR5 availability in the brain.^13, 33^

The reference region-based Logan method^34^ was used to calculate DVR (henceforth DVR' to distinguish from 2TCM results) in subjects without arterial sampling. The results of the Logan analysis were compared against the 2TCM results and were subsequently used to quantify [^11^C]ABP688 binding for all subjects.

DVR and DVR' were calculated for pre-defined brain regions including the following: dorsal caudate nucleus, dorsal putamen, ventral striatum, cingulate gyrus, hippocampus, amygdala, dorsolateral prefrontal cortex, orbital prefrontal cortex, medial prefrontal cortex temporal lobe, and parietal lobe.

### SPM analysis

A voxel-wise comparison of mGluR5 availability between the two cohorts was also performed using Statistical Parametric Mapping (SPM5).^35^ Parametric images of DVR' were generated for all subjects using the Logan method with the gray matter of the cerebellum as reference region. The voxel maps were co-registered to the corresponding MRI images as described above. Each MRI was transformed into a high-resolution template space Montreal Neurological Institute space using the Advanced Normalization Tools package.^36^ Using the PET-to-MRI co-registration transformation and nonrigid MRI transformation, all voxel maps were brought into the high-resolution template space. Voxel maps were smoothed using an 8-mm Gaussian kernel. A two-sample *t*-test was used to assess differences between MDD patients and healthy volunteers. No global normalization or grand mean scaling was used. Significant differences between the groups were accepted at *α*=0.05 after Gaussian random field correction.

### Symptomatology assessments

The following psychiatric scales were administered at the time of screening (within 3 weeks of the PET scan) to confirm eligibility for the study: CGI-S, Hamilton Depression Scale (HAM-D), Montreal Cognitive Assessment (MoCA) and the MMSE. In addition to the above scales, within 1 day of the PET scan, the following scales were assessed in the depressed cohort only to evaluate correlations between psychiatric symptoms and regional variations in mGluR5 density: Geriatric Depression Scale (GDS), Beck Suicidal Ideation Scale (BSIS), Generalized Anxiety Disorder Severity Scale (GADSS), Hamilton Anxiety Scale (HAM-A), Penn State Worry Questionnaire (PSWQ), State Trait Anxiety Scale (STAS) and Symptom Checklist-90-Revised (SCL-90).

### Statistical analysis

Assuming normal data distribution and an *α* of 0.1 (two-sided) for this pilot study, a sample of 22 healthy volunteers and 22 patients with MDD would provide 80% power to show a statistically significant difference if the true differences in the means between the patient groups and the healthy volunteers is 6.6–10.1%. A difference of 10% between groups was considered to be of clinical interest.

The agreement between results of the 2TCM, using the population average metabolite values (or individual's values when available), and those of the Logan analysis was tested graphically by scatter plot between the two parameters, and quantitatively by Pearson product–moment correlation.

For the primary end point, a linear mixed model, taking into account center, brain region, side (left and right), sex and center-by-cohort, region-by-cohort, side-by-cohort and sex-by-cohort interactions, and the random effect of subject, was used to compare patient populations. The linear mixed effects model also incorporated standard errors of the *V*_T_ measurement as weights when *V*_T_ was used as the outcome. Within the same subject, the covariance structure among regions was modeled by compound symmetry and the covariance structure between sides was modeled by unstructured variance. *V*_T_ measurements were log-transformed before fitting the linear mixed effect model. Analysis was performed using SAS 9.3 (SAS Institute, Cary, NC, USA).

To determine differences in [^11^C]ABP688 binding between patients with early- and late-onset depression, an exploratory analysis of the MDD population was performed by fitting a linear mixed model with age of onset of depression (early (<50 years, *n*=16) versus late onset, *n*=4), center, brain region, side, center-by-age of onset, region-by-age of onset, side-by-age of onset, region-by-side-by-age of onset and center-by-side-by-age of onset interactions as fixed effects, and subject as the random effect (Sex effects were not included as they were not found to be significant in the primary analyses).

The Spearman's correlation coefficient between the full or extracted score on each of the psychiatric assessment scales and regional binding was also calculated.

## Results

Healthy volunteers (*N*=22) and patients with MDD (*N*=20) completed the study and were comparable in terms of baseline characteristics and demographics (Table 1).

### Analysis of imaging data

A 2TCM was used to calculate regional *V*_T_ values for 16 healthy volunteers (73%) and 14 patients with MDD (70%) for whom arterial blood samples were available. *V*_T_ in the gray matter of the cerebellum did not differ between healthy volunteers and patients with MDD. In general, the correlation coefficients (Table 1) and scatter plots for individual patients (data not shown) showed good agreement between DVR (2TCM) and DVR' (reference Logan method) with the average correlation between the two measures being 0.79 (0.08) and 0.84 (0.18), for healthy volunteers and MDD subjects, respectively. Therefore, DVR' values from the reference Logan analysis were used for most statistical analyses as this variable does not require an arterial input function and was available for all subjects (Table 2).

**Figure 1:**
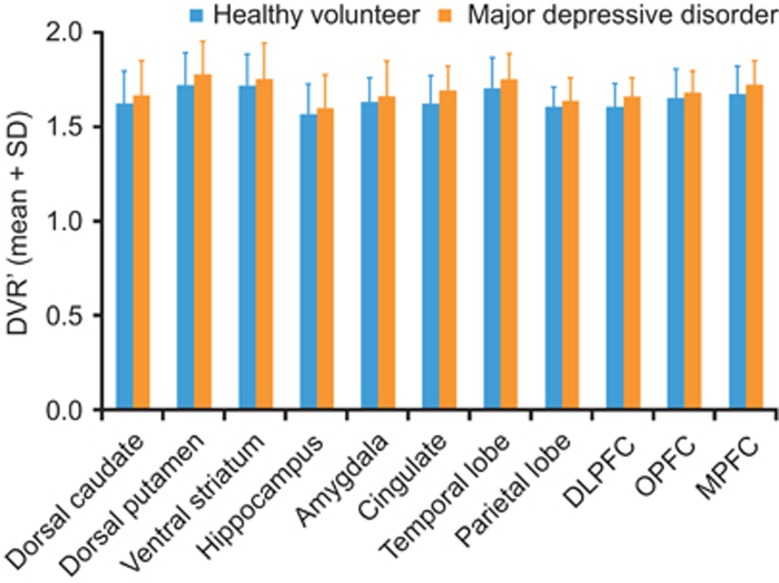
Comparison of DVR' values from different regions of the brain between elderly patients with major depressive disorder (*n*=22) and elderly healthy volunteers (*n*=20). DLPFC, dorsolateral prefrontal cortex; DVR', distribution volume ratio calculated using Logan; MPRF, medial prefrontal cortex; OPFC, orbital prefrontal cortex.

The mean regional DVR' values varied between 1.56 and 1.79. The lowest average DVR' values were obtained from the hippocampus and the highest were measured in the striatum (ventral striatum and putamen; Table 2 and Figure 1). Across regions, DVR' of depressed subjects was between 0.4 and 4.6% larger than the controls. Similarly, *V*_T_ of depressed subjects was between 7.2 and 24.3% greater than the controls. However, the linear mixed model indicated no significant group differences in [^11^C]ABP688 DVR' between healthy volunteers and patients with MDD in any brain region (Figure 1). Correspondingly, there were no significant group differences in *V*_T_ (in the reduced subject pool with arterial analysis) between healthy volunteers and patients with MDD. For both outcome measures (*V_T_* and DVR'), the linear mixed effects model showed a significant effect of center and region.

Results of the voxel-based analysis confirmed the findings of the primary reference region-based approach. Voxel-level SPM analysis found no regions within the brain where [^11^C]ABP688 DVR' was different between patients with MDD and healthy volunteers.

**Figure 2:**
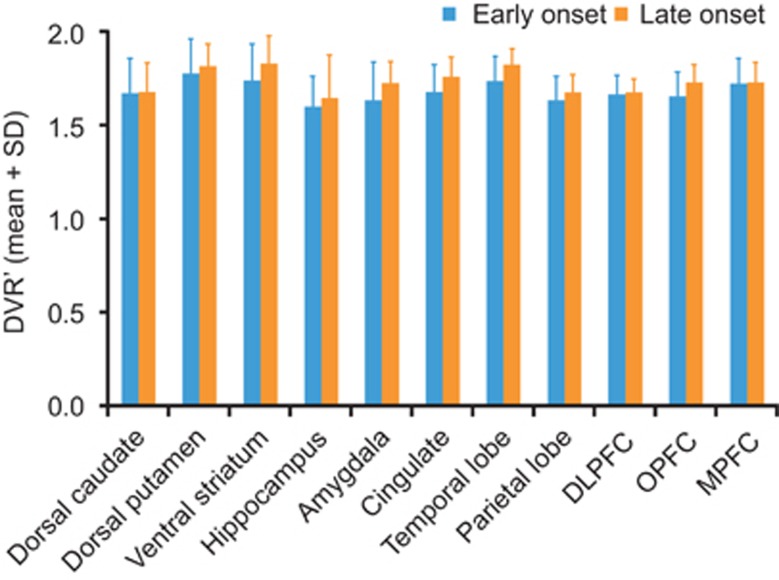
Comparison of DVR' values from different regions of the brain between elderly patients with major depressive disorder with early (*n*=16) and late (*n*=4) onset depression. DLPFC, dorsolateral prefrontal cortex; DVR', distribution volume ratio calculated using Logan; MPRF, medial prefrontal cortex; OPFC, orbital prefrontal cortex.

Of the 20 patients with MDD who took part in the study, 16 were considered to have early-onset of depression and four to have late-onset of depression. When patients with early- and late-onset of depression were compared, no differences were observed in DVR' of [^11^C]ABP688 in any brain region (Figure 2).

Exploratory analyses of cognitive and symptomatology assessments revealed significantly positive correlations between MMSE score and DVR' of [^11^C]ABP688 in the left ventral striatum, and the left and right caudate regions (Supplementary Material). Significant positive correlations were also found between MoCA score and DVR' values in the right dorsal caudate, and GDS score and DVR' values in the left hippocampus (Supplementary Material). No significant correlations were noted for any other symptom scale assessed (Supplementary Material). None of these results would survive correction for multiple comparisons.

### Safety assessments

[^11^C]ABP688 was generally well tolerated following single-dose administration in elderly patients with MDD and healthy volunteers. The overall incidence of adverse events was similar in patients with MDD (*n*=2; 10%) and healthy volunteers (*n*=1; 4.5%). All adverse events were classified as mild in severity, none were considered to be clinically significant and none were suspected to be related to study drug. No serious adverse event and no clinically relevant changes in laboratory assessments, vital signs or electrocardiogram were reported.

## Discussion

Metabotropic glutamate receptors, particularly mGluR5, have been implicated in antidepressant action and mood regulation.^7^ mGluR5 antagonists have been shown to have antidepressant effects;^8, 12, 37, 38^ therefore, an understanding of how mGluR5 availability varies in mood disorders may help devise appropriate treatment strategies. This is particularly important in the elderly, considering the widespread prevalence of MDD in this population, which is an increasing problem as the general population ages. The present study compared regional differences of mGluR5 availability in the brains of elderly patients with MDD and elderly healthy volunteers with comparable demographics, using PET and the radiotracer [^11^C]ABP688.

Studies have shown that [^11^C]ABP688 binding is altered following drug-induced perturbations of endogenous glutamate levels in humans,^39^ baboons^40^ and rats^41^ (although findings in rats are not entirely consistent^42, 43^), suggesting that this binding may be sensitive to glutamate variation. As [^11^C]ABP688 does not bind to the same site as glutamate on mGluR5, we and others have hypothesized that the presence of glutamate may alter [^11^C]ABP688 affinity for mGluR5.^39, 40, 41^ As magnetic resonance spectroscopy studies have reported higher levels of glutamate metabolites in the frontal cortex in depression (including late-life depression),^44^ this variability presents a potential confound for study results. However, metabolite changes were not consistently identified in other brain regions, and as the majority of metabolites measured by magnetic resonance spectroscopy are intracellular, it is difficult to know what, if any, effects on binding this would cause,^44^ especially on global binding as measured in this work. Further, despite potential intersubject variability in glutamate transmission, previous [^11^C]ABP688 studies in controls have not shown high variance in baseline binding.^24, 45^ Moreover, [^11^C]ABP688 has been successfully used previously to detect mGluR5-binding differences in depression, smoking and obsessive-compulsive disorder, and in response to antidepressant treatment.^14, 15, 46, 47^

In this study, we observed no statistically significant difference in [^11^C]ABP688 binding between elderly patients with MDD and elderly healthy volunteers. Moreover, radioligand binding was not found to differ between elderly patients with early-onset and late-onset depression. To our knowledge, this is the first study to evaluate mGluR5 in elderly depression. To improve generalizability, subjects were recruited from two sites. Linear mixed effects models run using either *V*_T_ or DVR' as the outcome measure indicated that there was a significant effect of center. This could be because of differing characteristics of the subjects recruited at each site or the characteristics of the PET imaging. However, on average (across all regions), the differences in DVR' binding between sites was 5.5±2.3% and 4.6±3.0% in the controls and depressed subjects, respectively (data not shown). Therefore, although significant, these small differences would not confound a biological signal and are negligible.

The optimal method to quantify [^11^C]ABP688 binding in the brain is 2TCM.^14^ However, this model requires an arterial input function. As we were unable to obtain an input function for all patients, we also used a reference region-based method to quantify [^11^C]ABP688 binding. As we have previously shown,^48^ binding estimates based on reference tissue modeling are biased compared with those estimated with an arterial input function. Further, group differences in binding in the reference region can confound depressed/control comparisons, as has been observed in other tracers.^49^ However, in this specific case, use of DVR' likely did not affect the ultimate conclusion as the binding in the reference region (*V*_T_, as assessed with arterial plasma) was not significantly different between groups and a depressed/control comparison (linear mixed effects model) performed only with subjects receiving arterial lines with both radioactivity and metabolite sampling (13 MDD and 11 controls) also yielded insignificant differences.

Deschwanden *et al.*^13^ found lower levels of mGluR5 binding in patients with MDD compared with healthy controls, and reduced mGluR5 protein expression in the prefrontal cortex of post-mortem brains of depressed subjects. In contrast, in this work, binding in the MDD group was higher than controls on a regional level (although differences did not reach significance), whereas voxel-level analysis yielded no cohort differences. There were several methodological differences between that study and ours, including radioligand administration. In our study, a single bolus, versus bolus plus infusion method, of the tracer was administered. However, these two methods have been shown to yield comparable results.^33^ Although bolus infusion techniques have the advantage of requiring fewer blood samples, potentially less time in the scanner and less computationally expensive modeling, we chose to use a bolus protocol because of the advantages of allowing accurate quantification of whole-brain binding (including regions with differing kinetics) and reduced dose.^50^ The SPM analysis used in the present study was also more conservative regarding significance levels and correction for multiple comparisons.^13^ However, the discrepancies between these two studies are most likely due to differences in patient populations (for example, age, severity of MDD and medication used) and imaging methods. In particular, the age range in that study was 22–59 years. As such, differing results may represent pathophysiological differences between depression in early and late adulthood (for example, mGluR5-mediated versus vascular disease).

Further, there is inconsistency in the literature regarding the role of mGluR5 in depression. A recent post-mortem study showed reduced GRM5 (which encodes mGluR5) in depressed subjects (in males only).^51^ However, in preclinical studies, mGluR5 knockout mice exhibit reduced depression-like behaviors and blockade of mGluR5 produces an antidepressant effect^52^ (which has also been shown in humans with mGluR5-negative allosteric modulators^53^). To address these inconsistencies and potential age effects, future studies will need to be performed.

Another potential area for further research is the correlation between MDD symptom severity and mGluR5 availability in different regions of the brain. In the present study, exploratory analyses revealed a positive correlation between GDS score and DVR' of [^11^C]ABP688 in the left hippocampus. In contrast, Deschwanden *et al.*^13^ reported the opposite tendency and a negative correlation between depressive symptoms, as assessed with the Beck Depression Inventory, and mGluR5 binding in all regions where DVR of [^11^C]ABP688 was decreased in MDD patients compared with controls (for example, in the parietal, temporal, frontal areas, thalamus and hippocampus). The discrepancy between the two studies may be worthy of further investigation.

No significant relationship was observed between the GADSS and binding in any region, despite the preclinical literature linking anxiety and mGluR5 (refs. 8, 54, 55, 56) and the use of mGluR5 antagonists as potential anxiolytics.^57^ However, the present study did demonstrate a significant positive correlation between MMSE score and DVR' of [^11^C]ABP688 in the left ventral striatum and bilateral caudate as well as between MoCA score and DVR' in right caudate. This is consistent with previous work showing reduced [^11^C]ABP688 binding in behavioral variant frontotemporal dementia. However, binding reductions in that work were widespread.^58^ Caution should be taken in the interpretation of these correlations as these results would not survive correction for multiple comparisons and therefore require further study. However, as this may suggest a link between mGluR5 and neurodegeneration (particularly given the vulnerability of mGluR5 to amyloidosis^59, 60^ and the presence of this and similar pathologies in geriatric populations^61, 62^), future studies designed to investigate the role of mGluR5 in cognitive functioning might be of interest.

In summary, results of the present study found no significant difference in mGluR5 density in the brain of elderly subjects with MDD compared with elderly healthy volunteers. Similarly, no significant difference in binding of [^11^C]ABP688 between patients with early- or late-onset depression was detected. Further work is required to determine whether this represents differences in the pathophysiology of depression in the elderly, an effect of age on mGluR5 or another mechanism. However, current results provide initial insight into the role of mGluR5 in elderly depression, a growing global problem.

## Figures and Tables

**Table 1:** Demographic summary by study group (total population)

	*Patients with MDD (*N=*20)*	*Healthy volunteers (*N=*22)*	*Total (*N=*42)*	P-*value*
*Age (years)*				
Mean (s.d.)	63.0 (6.33)	66.4 (7.30)	64.8 (6.98)	0.120^a^
Range	55–80	55–78	55–80	

*Gender, n (%)*				
Female	15 (75.0)	13 (59.1)	28 (66.7)	0.275^b^
Male	5 (25.0)	9 (40.9)	14 (33.3)	

*Race, n (%)*				
Caucasian	13 (65.0)	17 (77.3)	30 (71.4)	—
Black	6 (30.0)	4 (18.2)	10 (23.8)	
Asian	1 (5.0)	0 (0.0)	1 (2.4)	
Other	0 (0.0)	1 (4.5)	1 (2.4)	

*Weight (kg)*				
Mean (s.d.)	83.18 (17.908)	81.02 (14.052)	82.05 (15.841)	—
Range	61.6–122.9	62.5–116.6	61.6–122.9	

*Height (cm)*				
Mean (s.d.)	166.7 (9.64)	168.6 (8.09)	167.7 (8.81)	—
Range	149–180	156–180	149–180	

Abbreviation: MDD, major depressive disorder.

a *P*-value is from a two-sample *t*-test.

b *P*-value is from a *X*^2^-test.

**Table 2:** DVR (2TCM method) and DVR' values (Logan method) for each brain region (pharmacokinetic analysis population)

	*DVR (2TCM method)*		*DVR' (Logan method)*	
	*HV* (N=*16*)	*MDD* (N=*14*)	*HV* (N=*22*)	*MDD* (N=*20*)
*Dorsal caudate*				
Left				
Mean (s.d.)	1.64 (0.28)	1.68 (0.29)	1.65 (0.19)	1.70 (0.18)
*P*-value^a^			<0.001	<0.001
Right				
Mean (s.d.)	1.60 (0.24)	1.65 (0.33)	1.61 (0.17)	1.64 (0.20)
*P*-value^a^			<0.001	<0.001

*Dorsal putamen*				
Left				
Mean (s.d.)	1.71 (0.26)	1.77 (0.27)	1.73 (0.19)	1.77 (0.18)
*P*-value^a^			<0.001	<0.001
Right				
Mean (s.d.)	1.71 (0.24)	1.80 (0.28)	1.74 (0.16)	1.79 (0.18)
*P*-value^a^			<0.001	<0.001

*Ventral striatum*				
Left				
Mean (s.d.)	1.78 (0.27)	2.02 (0.70)	1.74 (0.21)	1.79 (0.20)
*P*-value^a^			<0.001	0.748
Right				
Mean (s.d.)	1.71 (0.23)	1.81 (0.36)	1.70 (0.16)	1.73 (0.21)
*P*-value^a^			<0.001	<0.001

*Hippocampus*				
Left				
Mean (s.d.)	1.67 (0.31)	1.73 (0.27)	1.58 (0.16)	1.63 (0.19)
*P*-value^a^			0.020	<0.001
Right				
Mean (s.d.)	1.62 (0.24)	1.67 (0.36)	1.56 (0.16)	1.57 (0.20)
*P*-value^a^			<0.001	<0.001

*Amygdala*				
Left				
Mean (s.d.)	1.69 (0.22)	1.70 (0.34)	1.64 (0.13)	1.65 (0.20)
*P*-value^a^			<0.001	<0.001
Right				
Mean (s.d.)	1.71 (0.23)	1.73 (0.32)^b^	1.65 (0.14)	1.67 (0.18)
*P*-value^a^			0.001	<0.001

*Cingulate*				
Left				
Mean (s.d.)	1.62 (0.22)	1.74 (0.28)	1.62 (0.16)	1.69 (0.16)
*P*-value^a^			<0.001	<0.001
Right				
Mean (s.d.)	1.63 (0.22)	1.70 (0.22)	1.64 (0.15)	1.69 (0.12)
*P*-value^a^			<0.001	<0.001

*Temporal lobe*				
Left				
Mean (s.d.)	1.74 (0.23)	1.78 (0.25)	1.70 (0.16)	1.75 (0.14)
*P*-value^a^			<0.001	<0.001
Right				
Mean (s.d.)	1.76 (0.23)	1.80 (0.23)	1.72 (0.16)	1.76 (0.14)
*P*-value^a^			<0.001	<0.001

*Parietal lobe*				
Left				
Mean (s.d.)	1.59 (0.17)	1.64 (0.23)	1.60 (0.11)	1.63 (0.13)
*P*-value^a^			0.006	<0.001
Right				
Mean (s.d.)	1.60 (0.17)	1.64 (0.22)	1.61 (0.11)	1.65 (0.12)
*P*-value^a^			0.007	<0.001

*DLPFC*				
Left				
Mean (s.d.)	1.60 (0.20)	1.66 (0.20)	1.60 (0.13)	1.66 (0.11)
*P*-value^a^			<0.001	<0.001
Right				
Mean (s.d.)	1.62 (0.20)	1.66 (0.19)	1.62 (0.13)	1.67 (0.10)
*P*-value^a^			<0.001	<0.001

*OPFC*				
Left				
Mean (s.d.)	1.67 (0.21)	1.69 (0.20)	1.65 (0.15)	1.68 (0.11)
*P*-value^a^			<0.001	<0.001
Right				
Mean (s.d.)	1.68 (0.21)	1.70 (0.22)	1.67 (0.14)	1.68 (0.13)
*P*-value^a^			<0.001	0.001

*MPFC*				
Left				
Mean (s.d.)	1.68 (0.21)	1.74 (0.25)	1.69 (0.14)	1.73 (0.13)
*P*-value^a^			<0.001	<0.001
Right				
Mean (s.d.)	1.68 (0.23)	1.71 (0.24)	1.68 (0.16)	1.71 (0.14)
*P*-value^a^			<0.001	<0.001

Abbreviations: DLPFC, dorsolateral prefrontal cortex; DVR, distribution volume ratio; HV, healthy volunteers; MDD, major depressive disorder; MPRF, medial prefrontal cortex; OPFC, orbital prefrontal cortex; 2TCM, two-tissue compartment model.

a *P*-values based on Pearson product–moment correlation coefficients between DVR and DVR'.

b *N*=13, as for one subject the model did not converge.

## Notes

DPD serves on the advisory board of AbbVie and Lundbeck. JJM receives royalties for commercial use of the C-SSRS from the Research Foundation for Mental Hygiene. The remaining authors declare no conflict of interest.
