# Supplementary material for: Characterization of brain mGluR5 binding in a pilot study of late-life major depressive disorder using positron emission tomography and [11C]ABP688
Source: Transl Psychiatry. 2015 Dec 8;5(12):e693–. doi: 10.1038/tp.2015.189 (PMC5068588; doi:10.1038/tp.2015.189)
Supplement: Supplementary Information [file tp2015189x1.docx]

**Supplementary material: Correlation between DVR’ and scores on each of the individual psychiatric scales in the MDD patient population**

| **Scale** | **GDS** | | **HAM-D** | | **GADSS** | | **BSIS** | | **CGI** | | **HAM-A** | | **PSWQ** | | **SCL-90-R** | | **MoCA** | | **MMSE** | |  |
| --- | --- | --- | --- | --- | --- | --- | --- | --- | --- | --- | --- | --- | --- | --- | --- | --- | --- | --- | --- | --- | --- |
| **Mean (SD) score, Day 1***  **Range*** | 20.8 (5.47)  10–28 | | 20.6 (3.08)  17–30 | | 12.4 (3.99)  4–23 | | 2.0 (5.04)  0–20 | | 4.3 (0.44)  4–5 | | 18.8 (4.05)  11–27 | | 39.1 (10.94)  19–56 | | 96.7 (39.83)  38–169 | | 28.3 (1.25)  26–30 | | 29.0 (1.32)  26–30 | |  |
| **Brain region** | **Left** | **Right** | **Left** | **Right** | **Left** | **Right** | **Left** | **Right** | **Left** | **Right** | **Left** | **Right** | **Left** | **Right** | **Left** | **Right** | **Left** | **Right** | **Left** | **Right** | |
| **Dorsal caudate** |  |  |  |  |  |  |  |  |  |  |  |  |  |  |  |  |  |  |  |  | |
| Correlation coefficient^†^ | 0.239 | 0.362 | 0.053 | 0.326 | -0.068 | -0.214 | 0.127 | 0.105 | 0.030 | 0.190 | -0.050 | 0.219 | -0.118 | 0.148 | 0.293 | 0.294 | 0.349 | 0.491 | 0.461 | 0.527 | |
| p-value^‡^ | 0.311 | 0.116 | 0.825 | 0.161 | 0.777 | 0366 | 0.593 | 0.660 | 0.900 | 0.422 | 0.834 | 0.354 | 0.619 | 0.532 | 0.209 | 0.208 | 0.131 | **0.028** | **0.041** | **0.017** | |
| **Dorsal putamen** |  |  |  |  |  |  |  |  |  |  |  |  |  |  |  |  |  |  |  |  | |
| Correlation coefficient^†^ | 0.293 | 0.416 | 0.151 | 0.263 | 0.294 | 0.084 | 0.105 | -0.007 | 0.210 | 0.290 | 0.114 | 0.187 | -0.084 | 0.064 | 0.351 | 0.313 | 0.163 | 0.109 | 0.164 | 0.151 | |
| p-value^‡^ | 0.209 | 0.068 | 0.525 | 0.263 | 0.208 | 0.726 | 0.660 | 0.975 | 0.374 | 0.214 | 0.633 | 0.431 | 0.726 | 0.788 | 0.130 | 0.179 | 0.492 | 0.648 | 0.489 | 0.526 | |
| **Ventral striatum** |  |  |  |  |  |  |  |  |  |  |  |  |  |  |  |  |  |  |  |  | |
| Correlation coefficient^†^ | 0.364 | 0.044 | 0.336 | -0.002 | 0.071 | 0.271 | 0.178 | 0.160 | 0.170 | 0.411 | 0.225 | 0.088 | 0.164 | 0.154 | 0.234 | 0.338 | 0.277 | 0.115 | 0.492 | -0.062 | |
| p-value^‡^ | 0.115 | 0.856 | 0.148 | 0.995 | 0.765 | 0.248 | 0.453 | 0.499 | 0.473 | 0.072 | 0.341 | 0.712 | 0.489 | 0.518 | 0.321 | 0.145 | 0.237 | 0.630 | **0.027** | 0.794 | |
| **Hippocampus** |  |  |  |  |  |  |  |  |  |  |  |  |  |  |  |  |  |  |  |  | |
| Correlation coefficient^†^ | 0.450 | 0.349 | 0.201 | 0.313 | 0.132 | -0.082 | 0.095 | 0.027 | 0.110 | 0.250 | -0.072 | 0.198 | -0.104 | 0.119 | 0.367 | 0.258 | -0.034 | 0.338 | 0.248 | 0.383 | |
| p-value^‡^ | **0.047** | 0.132 | 0.396 | 0.178 | 0.578 | 0.731 | 0.692 | 0.910 | 0.644 | 0.287 | 0.763 | 0.402 | 0.663 | 0.617 | 0.111 | 0.272 | 0.886 | 0.146 | 0.291 | 0.095 | |
| **Amygdala** |  |  |  |  |  |  |  |  |  |  |  |  |  |  |  |  |  |  |  |  | |
| Correlation coefficient^†^ | 0.270 | 0.164 | -0.057 | 0.039 | -0.024 | 0.071 | -0.028 | -0.028 | 0.070 | 0.230 | 0.039 | 0.094 | -0.313 | -0.139 | 0.243 | 0.235 | 0.061 | 0.388 | 0.114 | 0.173 | |
| p-value^‡^ | 0.250 | 0.490 | 0.810 | 0.870 | 0.919 | 0.765 | 0.907 | 0.907 | 0.796 | 0.329 | 0.871 | 0.693 | 0.179 | 0.558 | 0.302 | 0.319 | 0.797 | 0.091 | 0.633 | 0.466 | |
| **Cingulate** |  |  |  |  |  |  |  |  |  |  |  |  |  |  |  |  |  |  |  |  | |
| Correlation coefficient^†^ | 0.390 | 0.388 | 0.221 | 0.105 | 0.160 | 0.050 | 0.186 | 0.008 | 0.290 | 0.250 | 0.108 | 0.061 | 0.027 | -0.091 | 0.408 | 0.403 | 0.029 | 0.244 | 0.228 | 0.299 | |
| p-value^‡^ | 0.089 | 0.091 | 0.348 | 0.660 | 0.501 | 0.834 | 0.434 | 0.972 | 0.214 | 0.287 | 0.651 | 0.799 | 0.910 | 0.702 | 0.074 | 0.078 | 0.904 | 0.299 | 0.333 | 0.201 | |
| **Temporal lobe** |  |  |  |  |  |  |  |  |  |  |  |  |  |  |  |  |  |  |  |  | |
| Correlation coefficient^†^ | 0.124 | 0.093 | -0.023 | -0.036 | 0.052 | 0.121 | -0.034 | 0.141 | 0.150 | 0.290 | -0.131 | 0.018 | -0.002 | -0.023 | 0.275 | 0.243 | 0.018 | 0.065 | 0.030 | 0.017 | |
| p-value^‡^ | 0.604 | 0.696 | 0.923 | 0.880 | 0.826 | 0.612 | 0.886 | 0.553 | 0.527 | 0.214 | 0.583 | 0.939 | 0.995 | 0.925 | 0.240 | 0.302 | 0.940 | 0.785 | 0.899 | 0.944 | |
| **Parietal lobe** |  |  |  |  |  |  |  |  |  |  |  |  |  |  |  |  |  |  |  |  | |
| Correlation coefficient^†^ | 0.168 | 0.127 | 0.137 | 0.045 | -0.008 | 0.052 | -0.007 | -0.069 | 0.381 | 0.411 | 0.055 | 0.019 | 0.158 | 0.179 | 0.347 | 0.259 | 0.110 | 0.087 | 0.009 | -0.066 | |
| p-value^‡^ | 0.478 | 0.595 | 0.564 | 0.850 | 0.975 | 0.826 | 0.975 | 0.774 | 0.098 | 0.072 | 0.817 | 0.937 | 0.505 | 0.449 | 0.133 | 0.269 | 0.644 | 0.716 | 0.971 | 0.781 | |
| **DLPFC** |  |  |  |  |  |  |  |  |  |  |  |  |  |  |  |  |  |  |  |  | |
| Correlation coefficient^†^ | 0.008 | 0.086 | -0.131 | 0.012 | -0.214 | 0.052 | 0.058 | 0.142 | 0.030 | 0.230 | -0.055 | -0.006 | 0.137 | 0.271 | 0.051 | 0.216 | 0.095 | 0.132 | 0.103 | 0.103 | |
| p-value^‡^ | 0.975 | 0.717 | 0.582 | 0.959 | 0.366 | 0.826 | 0.807 | 0.551 | 0.900 | 0.329 | 0.819 | 0.980 | 0.564 | 0.247 | 0.830 | 0.361 | 0.689 | 0.579 | 0.6665 | 0.665 | |
| **OPFC** |  |  |  |  |  |  |  |  |  |  |  |  |  |  |  |  |  |  |  |  | |
| Correlation coefficient^†^ | 0.032 | 0.064 | 0.069 | 0.031 | 0.177 | 0.328 | -0.101 | -0.140 | 0.311 | 0.210 | 0.204 | 0.178 | 0.208 | 0.103 | 0.219 | 0.252 | 0.050 | 0.021 | -0.206 | -0.213 | |
| p-value^‡^ | 0.894 | 0.787 | 0.773 | 0.895 | 0.455 | 0.158 | 0.671 | 0.556 | 0.183 | 0.374 | 0.388 | 0.454 | 0.379 | 0.665 | 0.354 | 0.284 | 0.833 | 0.930 | 0.384 | 0.367 | |
| **MPFC** |  |  |  |  |  |  |  |  |  |  |  |  |  |  |  |  |  |  |  |  | |
| Correlation coefficient^†^ | 0.396 | 0.362 | 0.218 | 0.159 | -0.036 | 0.154 | 0.186 | 0.093 | 0.370 | 0.290 | 0.222 | 0.140 | 0.032 | -0.020 | 0.183 | 0.232 | 0.251 | 0.187 | 0.336 | 0.231 | |
| p-value^‡^ | 0.084 | 0.116 | 0.357 | 0.502 | 0.879 | 0.516 | 0.434 | 0.697 | 0.108 | 0.214 | 0.348 | 0.555 | 0.892 | 0.932 | 0.441 | 0.326 | 0.285 | 0.430 | 0.147 | 0.326 | |

*Summary scores for each psychiatric scale based on the safety analysis population (N = 20, except for MoCA where N = 19). ^†^Estimated Spearman's correlation coefficients between each pair of psychiatric scale and pharmacokinetic derived primary endpoint of [^11^C]ABP688 binding calculated in each brain region; based on pharmacokinetic analysis population. ^‡^P-values based on pharmacokinetic analysis population. GDS, Geriatric Depression Scale; HAM-D, Hamilton Depression Scale; GADSS, Generalized Anxiety Disorder Severity Scale; BSIS, Beck Suicidal Ideation Scale; CGI, Clinical Global Impression Scale; HAM-A Hamilton Anxiety Scale; PSQQ, Penn State Worry Questionnaire; SCL-90-R, Symptom Checklist-90-Revised; MoCA, Montreal Cognitive Assessment; MMSE, Mini-Mental State Examination
